# Influence of Three Laser Wavelengths with Different Power Densities on the Mitochondrial Activity of Human Gingival Fibroblasts in Cell Culture

**DOI:** 10.3390/life13051136

**Published:** 2023-05-06

**Authors:** Agnieszka Nowak-Terpiłowska, Joanna Zeyland, Magdalena Hryhorowicz, Paweł Śledziński, Marzena Wyganowska

**Affiliations:** 1 Department of Biochemistry and Biotechnology, Poznan University of Life Sciences, 60-632 Poznan, Poland; agnieszka.terpilowska@up.poznan.pl (A.N.-T.); joanna.zeyland@up.poznan.pl (J.Z.); magdalena.hryhorowicz@up.poznan.pl (M.H.); 2 Department of Genome Engineering, Institute of Bioorganic Chemistry, Polish Academy of Science, 61-704 Poznań, Poland; psledzinski@ibch.poznan.pl; 3 Department of Dental Surgery, Periodontology and Oral Mucosa Diseases, Poznan University of Medical Sciences, 60-812 Poznan, Poland

**Keywords:** gingival fibroblasts, cell culture, low-level laser therapy, proliferation

## Abstract

Phototherapy plays a key role in wound healing and tissue regeneration. The use of lasers has the potential to become an effective and minimally invasive treatment in periodontal and peri-implant disease. The aim of this study was to evaluate the influence of three laser wavelengths with the combination of parameters such as power density and energy density on human gingival fibroblasts (hGFs) in vitro culture. Isolated cells were seeded in 96-well plates with culture medium (DMEM, Dulbecco’s modified Eagle’s medium) supplemented with 10% fetal bovine serum (FBS). After 24 h cells were irradiated (1064, 980 and 635 nm, various energy density value). After 24, 48 and 72 h, cells were evaluated for viability. Data were analyzed by ANOVA followed by Tukey’s HSD test. We found the best outcomes for hGFs irradiated with laser 1064 nm for all combinations of power output (50/400/1000 mW) and energy dose (3/25/64 J/cm^2^) after 48 h and 72 h compared with control group. Cell viability increase ranged from 0.6× (3 J/cm^2^, 50 mW) to 1.3× (64 J/cm^2^, 1000 mW). Our findings indicate that the appropriate use of low-level laser irradiation (LLLI) can increase the proliferation rate of cultured cells. The use of LLLI can be extremely useful in tissue engineering and regenerative medicine.

## 1. Introduction

Laser stands for light amplification by stimulated emission of radiation, which is strengthening light by forced emission radiation. Light contains a wide spectrum of frequencies and intensities. Laser light is consistent, monochromatic and focused because it includes only one frequency value (wavelength) and intensity. Light can be amplified, so its intensity can be increased by the stimulating emission techniques. Light treatment without any supporting scientific knowledge has a long history. The use of light therapy for certain specific somatic symptoms dates back to the 20th century. The theoretical basis for the structure of the laser was first elaborated by Albert Einstein (1917). It took 43 years for the American physicist Theodore Maiman to construct and patent the first working ruby laser. Nevertheless, the father of laser therapy was not Maiman, but Endre Mester—a Hungarian physician, who, since 1967, has published many works on the therapeutic use of ruby and helium–neon lasers. High and low energy lasers (also called biostimulation lasers) are now widely used in medicine, depending on the energy emitted. Therapy with low power laser (LLLT, low-level laser therapy) is well documented and confirmed by the results of more than hundreds of scientific studies conducted worldwide. An increasing number of meta-analyses conducted leave no shadow of doubt that the therapeutic application of LLLT in medicine can be effective. However, the need for further research on optimal characteristics (parameters) of LLLT is underlined [1,2,3,4].

Characterization and identification of factors affecting gingival fibroblasts proliferation is essential to facilitate tissue regeneration. The role of low-level laser irradiation (LLLI) on the proliferation of human gingival fibroblasts has not been well established. The role of LLLI in regenerative dentistry has not been sufficiently studied. There is still limited information regarding the impact of the low-level laser therapy (LLLT) on cells and the underlying biological mechanisms are poorly understood [5]. Laser therapy can enhance the proliferation rate of various cell types without causing any cytotoxic effects [6]. LLLT is of immense importance in the field of periodontology because of its potential associated with biostimulation, microbial inhibition, destruction and the recovery of soft and hard tissues, resulting in a beneficial therapeutic effect [7,8]. Laser irradiation parameters such as wavelength, laser output power and energy density influence the in vitro biostimulation effects at the cellular level. LLLT seems to be a promising tool in the treatment of wounds and tissue regeneration [9]. Because of this, it is particularly important to know the relevant combination of parameters to achieve the desirable outcomes in the treatment of patients [10].

The most popular laser wavelengths used for oral tissue treatment are described as red, infrared and blue light. Red lasers increase cellular activity and microcirulation, stimulate immune system by the activation of different leucocyte groups, increase fibroblasts viability and improve wound healing. Infrared lasers stimulate mitochondria and blue laser has antimicrobial and anti-inflammatory effects, increase blood circulation by increasing NO-release and improving tissue supply [11]. It is not surprising that beyond the clinical symptoms of a better gingival status (color, shape, and bleeding), it was observed that mitochondria changed after laser irradiation with the activation of various metabolic pathways and an increased production of ATP [10]. The structure of mitochondria can be different in different cell types of tissues. In living cells, mitochondria have a dynamic structure, can merge or divide. The mitochondria comprise about 10–15% of a living cell volume. Their main task is the production of ATP in the respiratory chain. It is well known that the cytochrome c oxidase complex, the final part of the mitochondrial respiratory chain, is absorbed in the red and infrared range, while in NADH complex (starter complex), is activated by blue light [12]. In the case of a diminished ATP synthesis, this leads to an overflow of the cell with calcium and activation of proteinases, resulting in the death of the cell—necrosis. According to current data, the low-level laser therapy in gingival tissue is effective only in a therapeutic window in which biostimulation occurs. When the dose of irradiation is insufficient, there is no effect of treatment. On the other hand, when the dose is higher than the optimal, it may inhibit biostimulation. It is therefore important to find the optimal irradiation parameters for a predictable protocol of biostimulation [13].

In our research, we used a mitochondrial activity as a marker of fibroblast vitality. Besides general effects on the irradiated tissue or cells, light is absorbed at the molecular level. It can activate or inhibit the biochemical processes. The different light length can activate the different complex of respiratory chain in cells. In this aspect, the red and blue ranges seem to be the most effective.

The aim of this study was to evaluate the influence of three laser wavelengths with a combination of parameters such as power density and energy density on human gingival fibroblasts in vitro culture. In order to analyze the effect of LLLT on the in vitro proliferation of gingival fibroblasts, we developed a primary culture of human gingival fibroblasts. We assessed whether irradiation with lasers of different wavelengths (1064, 980 and 635 nm) can influence wound healing by stimulating mitochondrial activity of fibroblasts.

## 2. Materials and Methods

### 2.1. Cell Culture—Gingival Fibroblasts Isolation and Cultivation

In this study, human gingival fibroblasts were isolated from gingival tissue obtained from five healthy patients during standard surgical procedure. This study was conducted with the approval of the bioethics committee (consent no. 150/17 from 2 March 2017 by decision of Bioethics Commission at the Medical University of Karol Marcinkowski in Poznan), and all subjects gave their informed consent for inclusion before they participated in the study. Gingival fibroblasts were transferred in aseptic conditions from freezing medium (Dulbecco’s modified Eagle’s medium (DMEM)/F12 (1:1), 10% FBS, 10% dimethyl sulfoxide (DMSO). The cell culture was maintained in DMEM/F12 medium (1:1) supplemented with 10% fetal bovine serum (FBS) and 1% antibiotic/antimycotic solution (50 μg/mL of gentamicin sulfate, 100 IU of penicillin and 50 μg/mL of streptomycin). Cultivation was conducted at the temperature of 37 °C at 5% CO_2_ content in a humid environment, in sterile conditions. The medium was replaced every 3 days. Cultures of 70–80% confluence were passaged by digestion in tripsin-EDTA solution (0.25% tripsin, 0.02% EDTA). 

Before laser irradiation, the cells were removed from TC-flasks by trypsinization, and a medium solution without supplementation was added to achieve a concentration of 5 × 10^4^ cells/mL. An amount of 100 μL of cell suspension was transferred to 96-well plates. Three plates were identified that corresponded to laser-treated samples of different wavelengths (groups 1–3). In the case of a laser of a given wavelength, different energy density parameters were also distinguished. There were also unexposed control samples on the fourth plate. Cultivation in 96-well plates was conducted 24 h before laser irradiation. To evaluate the effects of diode laser on fibroblasts, the cells were grown in regular culture medium for 24 h. Before laser irradiation, the cell culture medium was removed and replaced with 100 μL of PBS in each well. Cell suspensions exposed to different laser irradiation parameters and the non-irradiated controls were assessed using Scepter™ 2.0 Cell Counter.

### 2.2. Laser Irradiation Procedures

Regarding the dispersion of laser light, black solid base plates were used. Such plates are recommended for top reading fluorescent instrumentation because of their low background and minimized light scattering.

Irradiation was performed through the open top of the plate by using the contra-angled hand piece with a 3 mm diameter laser beam.

### 2.3. Experimental Groups

I. The following parameters for diode 1064 nm laser (SMARTS, Lasotronix, Poland) stimulation for point will be provided: frequency: CW (continuous wave); power:

(1) 3 J/cm^2^, 50 mW, time 18 s;
(2) 25 J/cm^2^, 400 mW, time 18 s;
(3) 64 J/cm^2^, 1000 mW, time 18 s;
(4) non-irradiated control group.

II. The following parameters for diode 980 nm laser (SMARTS, Lasotronix, Poland) stimulation for point will be provided: frequency: CW; power:

(1) 3 J/cm^2^ 50 mW, time 18 s;
(2) 25 J/cm^2^ 400 mW, time 18 s;
(3) 64 J/cm^2^ i 1000 mW, time 18 s;
(4) non-irradiated control group.

III. The following parameters for diode 635 nm laser (SMARTS, Lasotronix, Poland) stimulation for point will be provided: frequency: CW; power:

(1) 3 J/cm^2^ 50 mW, time 18 s;
(2) 25 J/cm^2^ 400 mW, time 18 s;
(3) non-irradiated control group.

After the laser irradiation procedure, the cells were cultivated under standard conditions for 24, 48 and 72 h. After the specified time of incubation, a cell viability assay was conducted.

Irradiation setup of experimental groups is shown in Table 1.

### 2.4. Cell Viability Assay (CCK-8 Assay)

After the laser irradiation procedure, the stimulation of mitochondrial activity of fibroblast cells was assessed using the Cell Counting Kit-8 (CCK-8) assay. Cell Counting Kit-8 allows sensitive colorimetric assays for the determination of the number of viable cells in the proliferation and cytotoxicity assays. Each one of the 96-well plates was measured at 24, 48 and 72 h after laser irradiation. The CCK-8 assay procedure was as follows. A total of 10 μL of CCK-8 was added to each well and the plate was incubated for 2 h at 37 °C. Each plate was directly scanned, and the absorbance was measured at 450 nm using a microplate reader Infinite M200 PRO (TECAN). The results of cell number measurements with CCK-8 assay were statistically analyzed and presented as columns.

## 3. Results

In the case of group 1, irradiated with 1064 nm laser wavelength, the cell viability measured by mitochondrial activity analysis increased at 48 h and 72 h after laser irradiation in comparison with the non-irradiated control cells. In the case of cells examined 48 h after irradiation, cell viability increased 0.6 times for parameters 3 J/cm^2^ at 50 mW, 0.9 times for parameters 25 J/cm^2^ at 400 mW and 1.3 times for parameters 64 J/cm^2^ at 1000 mW compared to the control. In the case of cells examined 72 h after irradiation, cell viability increased 0.2 times for parameters 3 J/cm^2^ at 50 mW, 0.6 times for parameters 25 J/cm^2^ at 400 mW and 0.6 times for parameters 64 J/cm^2^ at 1000 mW compared to the control.

In the case of group 2, (980 nm) cultures presented higher cell viability only in the case of parameter 64 J/cm^2^; 1000 mW at 72 h after laser irradiation, compared to the control (0.2-fold increase). In the case of the other parameters and incubation period, cell viability increase was not meaningful in comparison with the non-irradiated control group.

**Figure 1:**
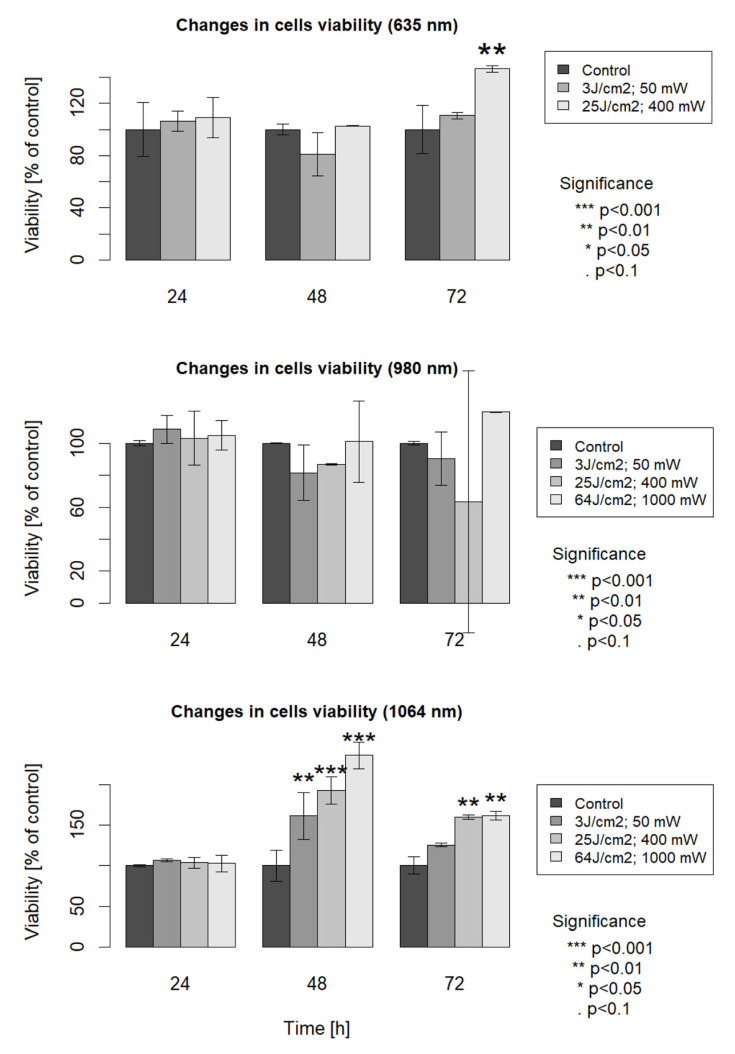
Analysis of cell viability (using mitochondrial activity analysis) measured at 24 h, 48 h and 72 h after irradiation with 635, 980 or 1064 nm laser wavelength. Data are expressed as a percentage of the non-irradiated control and the mean of pooled results from experiments performed in triplicate. Means were compared by ANOVA followed by Tukey’s HSD test. * *p* < 0.05, ** *p* < 0.01, *** *p* < 0.001 versus respective control (first bar in each panel, which represents the baseline level cells viability).

At 72 h after laser irradiation, the analysis of CCK-8 assays results showed that in group 3, irradiated with 635 nm, the cell viability increased 0.4 times only for parameter 25 J/cm^2^ at 400 mW. In the case of the other parameters and incubation periods, cell viability increase was not meaningful in comparison with the non-irradiated control group (Figure 1).

## 4. Discussion

Low-power lasers (LPL) have the same properties as high-power lasers (HPL), but their intensity is much weaker. The LLLT causes a slight increase in temperature (<1 °C) and is safe for healthy tissues in light therapy, affecting only pathologically changed areas. It is assumed that mechanisms of laser radiation are based on the resonance energy absorption by cells and concern cellular and molecular levels. The absorption effect is based on transmitting energy laser radiation to individual molecules of the organism remaining on the common energy level with photons. Absorption of laser radiation therefore causes an increase in total cell energy (internal energy). The receptor effect of biostimulation depends on the increase in cell metabolism as a result of absorbing laser light [14]. Total cell energy remains constant, and usable energy increases at the expense of internal energy. Hypothetical mechanisms of receptor and absorption effects are always present simultaneously.

Low power laser therapy is a supportive treatment used in physical therapy, pediatrics, dentistry, dermatology, general medical practice, acupuncture, addiction treatment, sports medicine, veterinary procedures and cosmetology. Low power laser light is a beam of photons which is absorbed by tissues and transforms in the mitochondria into intracellular energy [15].

LLLT affects numerous biological processes in the body, such as: (1) increasing the growth factors expression; (2) increasing the synthesis of ATP; (3) increasing cell proliferation and motility; (4) stimulates angiogenesis; (5) intensifies the remodeling of the extracellular matrix; (6) induces metabolic changes in selected neurotransmitters; (7) reduces nociceptive activity; and (8) modulates the immune system response [16,17,18,19]. The effects mentioned above concern various cell types, including macrophages, fibroblasts, endothelial cells, mast cells, osteoblasts, and mesenchymal stem cells (MSCs). To obtain the optimal therapeutic effect in particular diseases, the appropriate wavelength and the appropriate dose of energy that vary depending on the application should be used. A dose that is too high can cause adverse side effects, and too low will not cause any changes nor therapeutical influence. It also seems important to choose the right pulse emission at a specific frequency. All these abovementioned factors determine whether the laser energy reaches the tissues, and what their reaction to the treatment will be. Incorrect selection of parameters is one of the main causes of the ambiguous results of scientific studies. Poor scientific descriptions of procedures regarding the practical selection of the dosage amount in therapy confuse practitioners, and often leads to therapeutic errors and ineffective therapies.

The low-level laser therapy is recommended with the following parameters: power output within the mW range, energy dosage of 10^−2^–10^2^ J/cm^2^ and wavelengths of 450–1000 nm [20]. For example, a meta-analysis conducted by Zhao et al. showed that low power equal or less than 500 mW combined with energy density equal or greater than 5 J/cm^2^ could have an influence on reducing postoperative pain. LLLT can also positively affect wound healing in the case of gingival grafts [21]. To determine the most effective laser parameters, a postoperative analgesics administration should be eliminated during studies. Well-chosen parameters in the LLLT can improve therapy and reduce the free gingival graft (FGG) shrinkage in commonly applied periodontal procedure. It was observed that shrinkage of the FGG was higher in the control group than the test group in which a diode laser of the following parameters was applied: wavelength of 810 nm, power output of 10^−1^ W and energy density of 6 J/cm^2^ [22]. The abovementioned results are promising, but the laser parameters must result from a well-thought-out strategy, and so, more research is still needed to determine optimal laser settings.

In the present work, we evaluated lasers with three different wavelengths of 635 nm (the red spectrum), 980 nm (the near-infrared (NIR) spectrum) and 1064 nm (the infrared spectrum) to stimulate mitochondrial activity of human gingival fibroblasts (hGFs) in vitro. To assess mitochondrial activity of hGFs, we used an assay based on the ability of mitochondrial dehydrogenases to reduce WST-8 to formazan. Compared to other tests using different tetrazolium salts (MTT, XTT, MTS or WST-1), the detection sensitivity using CCK-8 is higher and toxicity is lower [23,24,25].

Photobiomodulation mediated by the red (635 nm) and the NIR (810 nm) light suggests that the biological response of the wound tissue depends on the wavelength employed. The effectiveness of 810 nm wavelength confirms findings from previous publications and, together with the partial effectiveness of 635 nm and the ineffectiveness of 730 and 980 nm wavelengths, can be explained by the absorption spectrum of cytochrome c oxidase, the candidate mitochondrial chromophore in LLLT. Photons in the red and the NIR can increase the accessibility of electrons that positively influences ATP generation and availability. The light-induced rise in intracellular cAMP promotes particular tissue repair and anti-inflammation mechanisms (i.e., cAMP inhibits TNF synthesis) [26].

Tissue biological response to phototherapy depends on the wavelength applied [27,28]. Positive effects were observed for NIR and especially for red light in different animal in vivo models. Low absorption of red and NIR wavelengths by water and tissue chromatophores enables deep penetration of lasers using red and NIR light [29], which allows the effective treatment of soft tissue wounds. Red light was shown to boost cytokines secretion in macrophages, while associated ATP accumulation stimulated collagen synthesis. The red LED light therapy significantly increased the expression of a transforming growth factor beta (TGF-β) protein related to tissue repair. An improvement of the dermo-epidermal junction was observed because of a growing density of collagen fibers [30]. Other studies have shown that LLLT can modulate inflammatory processes in periodontal tissue by influencing a local immune response and reducing LPS-stimulated PGE2 and IL-1b production in hGFs [31]. In studies of human periodontal ligament fibroblasts, laser irradiation (800 nm) reduced the expression of matrix metalloproteinase 8 (MMP8) protein, known to accelerate the degeneration of collagen in periodontal tissues. Applying LLLT in conservative periodontal treatments (calculus/microbial plaque removal) promotes wound healing and helps to avoid invasive treatments [32]. Light-induced analgesia is also linked to a reduction in inflammation. Lim et al. suggested not only direct but also indirect exposure to 635 nm light can inhibit activation of pro-inflammatory mediators in hGFs and may be clinically useful as an anti-inflammatory tool [33]. 

In the present study, we assessed mitochondrial activity of hGFs but not their anti-inflammatory activity. As mentioned, irradiation with 635 nm laser can inhibit activation of pro-inflammatory mediators and may have clinical applications. Increased mitochondrial activity enhances the generation of O^−^_2_, which can promote stem cell differentiation and cell growth depending on reactive oxygen species (ROS) concentration [34]. Molecular mechanisms of photobiomodulation therapy (PBM) are also connected with the endocannabinoid system. The use of AM281/AM630 antagonists of CBR1/CBR2 (cannabinoid receptor), respectively, significantly reversed the anti-inflammatory effect of the photobiomodulation therapy [35]. PBM is related to cannabinoid receptors activation by the ability to activate ATP-dependent K^+^ channels and p38 mitogen-activated protein kinase (MAPK) [36].

The potential use of 635 nm lasers in the promotion of cell growth and regeneration has been evaluated in osteoblasts and mesenchymal stromal cells [37]. As 635 nm laser irradiation support anti-inflammatory effects, it is not surprising that we did not observe significant changes for hGFs irradiated with the laser of red spectrum (635 nm) for all combinations of power output (50/400 mW) and energy dose (3/25 J/cm^2^) after 24 h, 48 h and 72 h compared with controls that were not irradiated. In our study, we observed the best results for hGFs irradiated with the laser of infrared spectrum (1064 nm) for all combinations of power output (50/400/1000 mW) and energy dose (3/25/64 J/cm^2^) after 48 h and 72 h compared with control that was not irradiated. Cell viability increase ranged from 0.6× (3 J/cm^2^, 50 mW) to 1.3× (64 J/cm^2^, 1000 mW). Our results remain opposite to the findings of Dilsiz et al. [38], who conducted a study on patients with Miller Class 1 and 2 recessions treated with SCTG and root-surface irradiation with Nd: YAG laser (1 W, 10 Hz, 100 mJ, 60 s, 1064 nm). The control group after SCGT without irradiation showed a greater reduction in recession depth and width compared with the irradiated group. These findings suggest, as mentioned before, that only the right parameter combination guarantees the success of phototherapy and further studies are required.

According to current knowledge, there is no established specific “therapeutic window” in which the biostimulation in periodontal tissue occurs. This therapeutic window means that the LLLT energy used is adequate for stimulated cell types. When the dose is insufficient, it does not produce any effect, and analogously when it is too high may draw bioinhibition. Our results agree with previous studies in which the best results were observed when the diode lasers in the 600–700 nm spectrum were effective in the 10 mW to 30 mW power range. The power range we used was higher, but the time of irradiation was shorter. Since a multitude of factors play a role in successful treatment, well-defined dosage guidelines can be made only for a specific laser [39]. The parameters used in experiment were connected with the type of device.

It is also worth considering the experiments using 3D cell cultures in vitro, which will not only allow us to study the depth of the penetration of laser light, but also well-reflect the mutual spatial relations between cells and the intercellular matrix.

## 5. Conclusions

We found the best outcomes for hFGs irradiated with laser 1064 nm for all combinations of power output (50/400/1000 mW) and energy dose (3/25/64 J/cm^2^) after 48 h and 72 h compared with the control group. Cell viability increase ranged from 0.6× (3 J/cm^2^, 50 mW) to 1.3× (64 J/cm^2^, 1000 mW). The appropriate use of LLLI can increase the proliferation rate of cultured cells, which can be especially useful in tissue engineering and regenerative medicine.

## Figures and Tables

**Table 1:** Irradiation setup of experimental groups. The frequency for all repetitions was constant, the exposure time was 18 s for all laser wavelengths and power densities; frequency: CW.

	Laser Type	Diode 1064 nm Laser	Diode 980 nm Laser	Diode 635 nm Laser
Power Density				
1		3 J/cm^2^, 50 mW	3 J/cm^2^, 50 mW	3 J/cm^2^, 50 mW
2		25 J/cm^2^, 400 mW	25 J/cm^2^, 400 mW	25 J/cm^2^, 400 mW
3		64 J/cm^2^, 1000 mW	64 J/cm^2^, 1000 mW	-
4		non-irradiated control	non-irradiated control	non-irradiated control

## Data Availability

The datasets generated during and/or analyzed during the current study are available from the corresponding authors upon reasonable request.
